# Ki-67 labeling in canine perianal glands neoplasms: a novel approach for immunohistological diagnostic and prognostic

**DOI:** 10.1186/1746-6148-9-83

**Published:** 2013-04-20

**Authors:** Rodrigo Storti Pereira, Augusto Schweigert, Guilherme Dias de Melo, Fernando Vissani Fernandes, Felipe Augusto Ruiz Sueiro, Gisele Fabrino Machado

**Affiliations:** 1 UNESP – Univ Estadual Paulista, College of Veterinary Medicine Araçatuba, São Paulo, Brazil; 2 School of Veterinary Medicine, Centro Universitário de Rio Preto (UNIRP) São José do Rio Preto, São Paulo, Brazil; 3 VETPAT – Laboratório de Patologia e Biologia Molecular Veterinária Campinas, São Paulo, Brazil; 4 Departamento de Clínica, Cirurgia e Reprodução Animal, Faculdade de Medicina Veterinária, UNESP – Universidade Estadual Paulista “Júlio de Mesquita Filho”, Rua Clóvis Pestana, 793, Araçatuba, SP CEP 16050-680, Brazil

**Keywords:** Cell proliferation, Computer-assisted image analysis, Dogs, Immunohistochemistry, MIB-1

## Abstract

**Background:**

The antibody Ki-67 is a reliable and easy tool to accurately assess the growth fraction of neoplasms in humans and animals, and it has been used to predict the clinical outcome. Therefore, the aim of the present study was to investigate the immunohistochemical expression pattern of Ki-67 in normal and neoplastic perianal glands of dogs to evaluate the possible use of this proliferation marker as an ancillary method of perianal tumor diagnosis. We studied 42 cases of perianal gland neoplasms including adenomas (n = 15), epitheliomas (n = 15), and carcinomas (n = 12). As controls, 13 tissue samples from normal perianal glands were used. A Ki-67 index was established by a computer-assisted image analysis and compared with manual counting.

**Results:**

Out of the 42 cases of perianal gland neoplasms, 34 were from males and eight from females. Recurrence was reported in 14 cases, being higher (8/12) in carcinomas. Immunostaining for Ki-67 revealed that the carcinomas showed a higher proliferation rate (9.87%) compared to groups of epitheliomas (2.66%) and adenomas (0.36%). For adenomas and epitheliomas of the perianal glands the computer-assisted counting and the manual counting gave similar results; however, only the computer-assisted image analysis was efficient to predict the perianal gland carcinoma recurrence.

**Conclusion:**

Since there were significant differences in the number of Ki-67-positive nuclei, this marker proved to be effective in helping the classification of perianal gland neoplasms and to refine the diagnosis criteria, especially in those samples with high variation in morphology/area. Also, higher Ki-67 index is related to recurrence in cases of perianal gland carcinomas. Further, the computer-assisted image analysis proved to be a fast and reliable method to assess the Ki-67 index in perianal gland neoplasms.

## Background

Perianal glands, also known as circumanal glands (by their location) and hepatoid glands (by their histological similarity with hepatocytes), are modified sebaceous glands present primarily in perianal skin [1].

Tumors of perianal glands are common in intact and aged male dogs. They occur occasionally in females and rarely in neutered males [2,3]. The causes of these neoplasms are unknown; however, gonadal hormones appear to interfere with perianal gland cell proliferation [1,3]. The possible influence of testosterone in perianal gland neoplasms is indicated by the high prevalence of benign tumor regression (up to 95%) after male neutering [1-4].

Adenoma is a common lesion in dogs and, besides hyperplasia, accounts for 8-10% of all canine skin tumors [4]. Adenomas generally retain the lobular architecture, presenting well-differentiated hepatoid cells and a single peripheral layer of basaloid reserve cells [1]. Some adenomas contain focal areas of pleomorphism that may suggest malignant transformation [5].

Several authors have grouped perianal gland epitheliomas with perianal gland adenomas [4,6], but the World Health Organization classified adenomas and epitheliomas in different categories [7]. Walder and Gross [8] classified the epitheliomas as low-grade perianal gland carcinomas. Distinction between adenomas and epitheliomas is easy when the basaloid reserve cells are organized in sheets, islands or trabeculae, together with moderate-low to moderate-high mitosis index, without atypia [1].

Perianal gland carcinomas are uncommon canine malignant neoplasms and occur almost exclusively in a perianal location. Well-differentiated carcinomas are clinically indistinguishable from adenomas. They are characterized by nodular formation. On the other hand, poorly differentiated carcinomas are poorly circumscribed and usually ulcerated [1]. The cells in well-differentiated carcinomas maintain the hepatoid morphology, forming solid islands and tightly packed trabaculae with a scarcity of collagen. The maturation patterns from reserve cells to hepatoid cells are disorganized and reserve cells are increased in number [1]. The distinction between perianal adenoma and carcinoma is often difficult: both may have poorly preserved lobulation and intermingling of germinal cells with mature hepatoid cells [3].

The Ki-67 antigen is expressed in G_1_, S, G_2_, and M phases of the cell cycle, but not in G_0_, and thus Ki-67 distinguishes between proliferating and quiescent cells [9]. Ki-67 expression is usually estimated as percentage of immunostained cells, with nuclear staining being the most common criterion for proliferative index [10]. Ki-67 is tightly controlled and regulated, implying a fundamental role in cell proliferation [11]. Further, the primary antibody anti-human Ki-67 (clone MIB-1) has been extensively used in studies to determine the prognosis of several types of canine neoplasms [12-14].

Although the morphological pattern of perianal gland neoplasms, when observed using hematoxylin and eosin stain (HE), is supposed to allow an accurate diagnosis, occasionally, the occurrence of focal pleomorphism in adenomas and epitheliomas can suggest malignant transformation, and result in recurrence. Therefore, the aim of this study was to investigate the immunohistochemical pattern of Ki-67 staining in normal and neoplastic canine perianal glands in order to evaluate the possible use of this proliferation marker as an ancillary method to determine perianal tumor diagnosis and prognosis.

## Methods

### Ethics

This study was approved by the Institutional Ethics and Animal Welfare Committee (Comissão de Ética no Uso de Animais - CEUA, UNESP, process number 02214-2011).

### Tissue samples

Forty-two canine perianal gland neoplasms were retrieved from the archives of a private practice laboratory of veterinary pathology (VETPAT – Campinas – São Paulo- Brazil). The owners were contacted and asked to answer a questionnaire regarding identification of the animals, evolution of the case, and recurrence. The specimens were re-evaluated by two pathologists. All specimens were removed during surgery, fixed in 10% neutral buffer formalin, embedded in paraffin, and consecutive sections were used for histology (HE) and immunohistochemistry.

The diagnosis of perianal gland neoplasia was recognized on HE-stained tissue sections following the criteria established by the World Health Organization (WHO) for glandular tumors of domestic animals [7]. The tumors examined included perianal gland adenomas (n *=* 15), epitheliomas (n *=* 15), and carcinomas (n = 12). All specimens were obtained from dogs having the neoplasm removed for the first time, i.e., no recurrent tumor was included. Normal perianal glands were obtained from dogs that were euthanized or died from other conditions (n *=* 13).

### Immunohistochemistry

5-μm thick sections were mounted on poly-L-lysine-coated slides, dewaxed, and rehydrated. Antigen retrieval was performed with citrate buffer (pH 6.0) in a microwave oven for 30 min. Endogenous peroxidase activity was blocked by incubating the sections in 2% (v/v) hydrogen peroxide (30 vol) diluted in 50% (v/v) methanol for 30 min. Subsequently, non-specific binding was blocked with 3% (w/v) non-fat dried milk in phosphate-buffered saline (PBS) pH 7.2 for 30 min. Sections were then incubated with the monoclonal mouse anti-human Ki-67 antibody (clone MIB-1, M7240, Dako), diluted 1:100 for 18–22 h at 4°C in a humidified chamber. The sections were subsequently washed in PBS and incubated with a biotinylated secondary antibody followed by the streptavidin-horseradish-peroxidase complex, in accordance with the manufacturer’s instructions (LSAB + Kit, K0690, Dako). The reaction was developed using 3,3'-diaminobenzidine (K3468, Dako). The slides were then counterstained with Harris’s hematoxylin, dehydrated, cleared, and mounted with coverslips. Negative controls were obtained by omitting the primary antibody. Positive controls included adjacent normal skin (basal cells) in each slide.

### Computer-assisted image analysis

**Figure 1:**
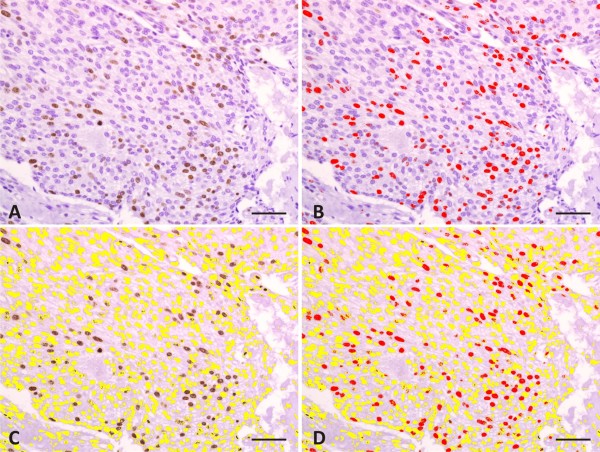
**Graphical representation of the method used to evaluate the Ki-67 index, quantified using the Image-Pro Plus 6.1 software.** (**A**) Photomicrograph of the selected microscope field exhibiting Ki-67-positive nuclei in a carcinoma hot spot. (**B**) Selection of the immunostained nuclei (in red). (**C**) Selection of the unstained nuclei (in yellow). (**D**) Measurement of the ratio of the immunostained nuclear area (in red) and the entire nuclear area, expressed as the percentage of the immunostained area. This method allows the rapid inclusion of all nuclei in the measurement and enables areas with tissue artifacts to be discounted. Scale bar = 50 μm.

**Figure 2:**
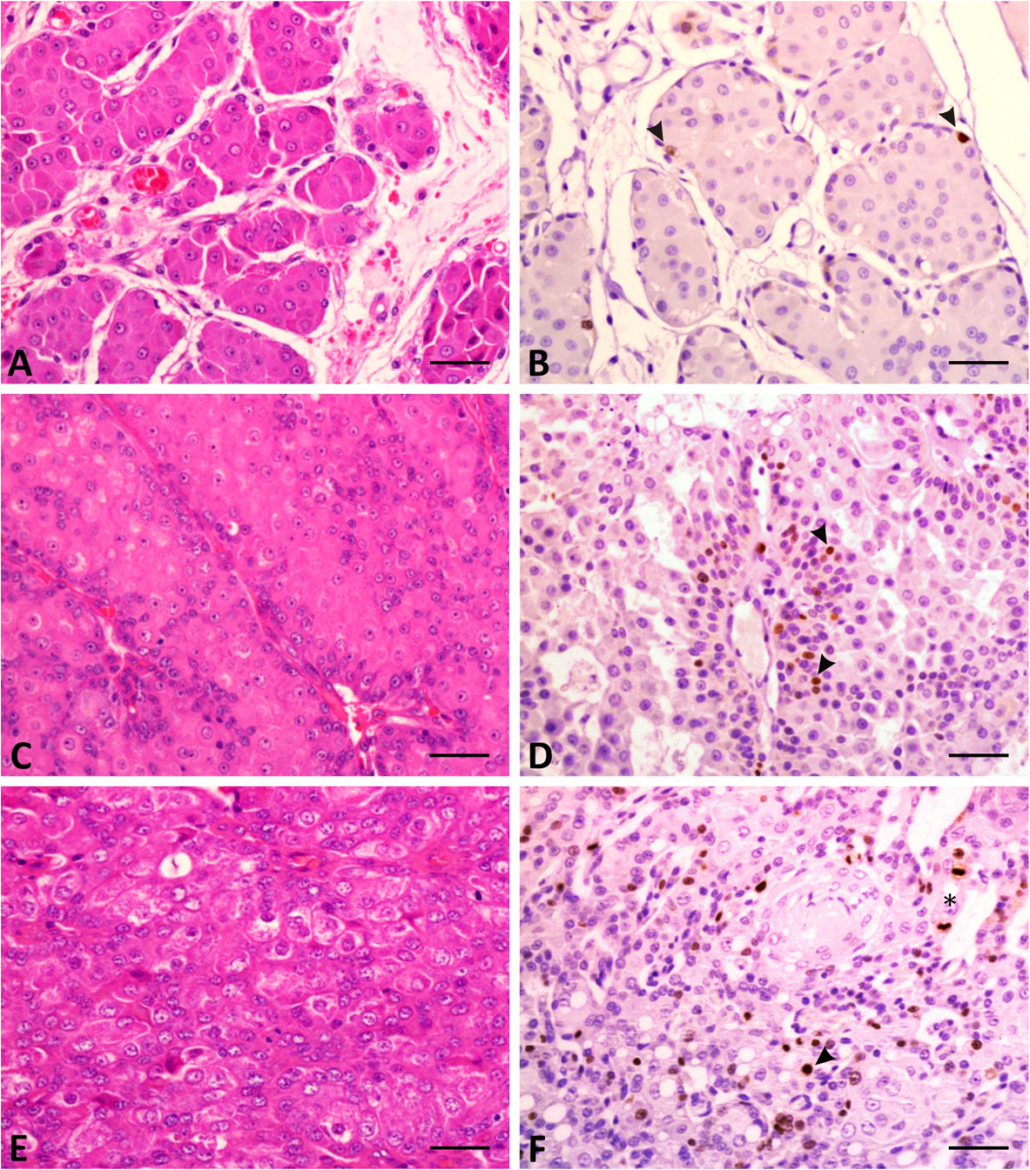
**Histopathological and immunohistochemical aspects of canine perianal gland neoplasms. A-B:** Perianal gland adenoma. Note the polyhedral cells grouped in nests and surrounded by a small amount of fibrous connective tissue (**A**) and the presence of rare Ki-67-positive nuclei (**B**) (*arrowhead*). **C-D:** Perianal gland epithelioma. Observe cells organized in nests with a thick basal layer (**C**) and with marked mitotic activity, evidenced by the Ki-67-positive staining (**D**) (*arrowhead*). **E-F:** Perianal gland carcinoma. Note the proliferation of polymorphic polyhedral cells organized in a solid pattern, with moderate anisokaryosis and anisocytosis (**E**), diffuse Ki-67-positive nuclear staining (*arrowhead*), and the presence of mitosis (*) (**F**). **A**, **C**, **E**: Hematoxylin and Eosin. **B**, **D**, **F**: Streptavidin-biotin peroxidase complex. Scale bar = 50 μm.

To determine the Ki-67 index, the whole slides were examined in low-power magnification (×100) to detect the areas with evident staining (“hot spots”), and pictures were taken in three representative high-power microscope fields (×400), in a total area of 271,518.1 μm^2^, randomly chosen across regions with the highest amount of Ki-67-positive cells (as illustrated in Figure 1A and Figure 2B, D, F), avoiding areas of inflammation, necrosis, and tissue artifacts [12-14]. The choice of the “hot spots” [15] was based on the assumption that the areas with a high proliferation index potentially predict a more aggressive biological behavior of the neoplasm. The counting of immunostained nuclei was performed colorimetrically, quantifying the percentage of the immunostained nuclear area in relation to the total nuclear area (Figure 1), based on the guidelines described by Melo and Machado [16], using the software Image-Pro Plus 6.1 (Media Cybernetics). Ki-67 scoring was also performed by manual counting by two pathologists, in the same areas as the computer-assisted analysis, in order to compare these two methods. All the analyses were performed blind, without knowledge of the experimental groups.

### Statistical methods

The differences among groups were determined by the Kruskal-Wallis test followed by Dunn’s multiple comparison test. For recurrence analyses, the Mann Whitney test was used. A value of P < 0.05 was considered statistically significant. Data are expressed as the median (minimum-maximum). A ROC (receiver operating characteristic) curve was used to determine the cut-off value for recurrence in perianal gland carcinomas, using the Ki-67 index obtained from the computer-assisted image analysis. All statistical analyses were performed using Prism 6 software (GraphPad).

## Results

Of the 42 neoplasms, 34 were from males and eight from females. Most of them were not neutered/spayed. The age ranged from 3–15 years and the evolution varied between 4 and 36 months (Table 1). Individual data from each animal may be found in Additional file 1: Table S1.

**Table 1:** Data from dogs included in the experimental groups

**Group**	**Total of cases**	**Gender**		**Age range**	**Spaying**		Follow-up* (months)	**Recurrence (n° of cases)**
		**Male**	**Female**	**(years)**	**Male**	**Female**		
Adenoma	15	11	4	5 - 13	0	3	8 - 24	3
Epithelioma	15	14	1	8 - 15	2	0	6 - 36	3
Carcinoma	12	9	3	3 - 12	0	1	4 - 24	8
Control	13	7	6	3-12	0	0	-	-

* time elapsed from the detection of the perianal nodule by the owner to the surgery (nodulectomy).

The adenomas were characterized by the presence of epithelial cells with abundant and eosinophilic cytoplasm, low pleomorphism, and basal cells with small and hyperchromatic nuclei. The cells were organized in islands separated by well-vascularized connective tissue (Figure 2A).

The epitheliomas were organized in lobules made up of epithelial cells with abundant and eosinophilic cytoplasm and low pleomorphism. The presence of basaloid cells with large nucleus, loose chromatin, evident nucleolus, and occasional mitosis was noted. Cell islands separated by connective tissue were also present (Figure 2C). In some samples, focal areas of squamous metaplasia were noted, and also areas with high-grade anaplasia, suggesting malignant transition.

In carcinomas, the noticed proliferation pattern was cribriform. The cells presented a large and clear nucleus, loose chromatin, evident nucleolus, cytoplasm with poorly defined borders, and frequent mitosis (Figure 2E).

**Figure 3:**
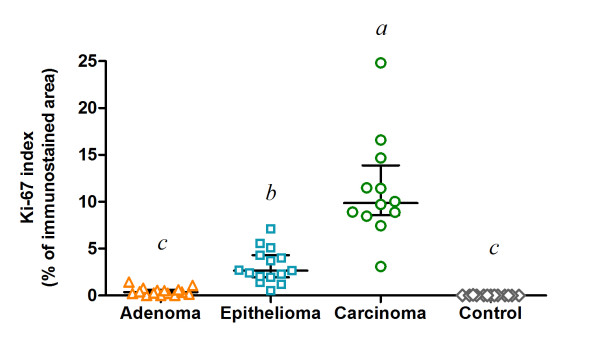
**Scatter plot showing the Ki-67 index assessed by computer-assisted image analysis (percentage of immunostained nuclear area in relation to the total nuclear area), in different types of canine perianal gland neoplasms: adenoma (n = 15), epithelioma (n = 15), and carcinoma (n = 12), and in control dogs (n = 13).** Horizontal lines indicate the median and the interquartile range. ^abc^ Groups with no common superscript letter differ significantly by Dunn’s multiple comparison test (P < 0.05).

In canine normal skin sections used as controls, Ki-67 was observed only in the *stratum basale*. By means of computer-assisted image analysis, the normal perianal glands showed a Ki-67 index of 0.00% (0.00-0.07); the only positive cells were detected among basaloid reserve cells. The Ki-67 index from adenomas corresponded to 0.36% (0.00-1.43), related to only a few scattered cells (Figure 2B). Epitheliomas showed positive staining mostly in basaloid cells with a Ki-67 index of 2.66% (0.54-7.10); in some cases the stained cells presented a rosette pattern (Figure 2D). Finally, carcinomas presented the highest Ki-67 index, 9.87% (3.09-24.82), and the stained cells were diffusely distributed throughout the tissue (Figure 2F). A significant difference was observed in the Ki-67 indexes among adenomas, epitheliomas and carcinomas (P < 0.0001), but not between the adenomas and the normal glands (Figure 3).

**Figure 4:**
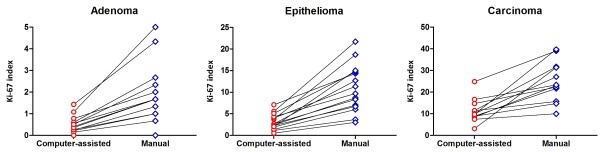
**Comparisons between the computer-assisted counting and the manual counting to establish the Ki-67 index in perianal gland adenomas, epitheliomas, and carcinomas.** The lines connect the values of each method in the same dog.

The Ki-67 index obtained from the manual counting was also compared among the neoplasms (Additional file 1: Figure S1): the Ki-67 index of carcinomas was 23.17% (10.00-39.67), with no difference compared to epitheliomas, which showed a Ki-67 index of 9.67% (3.00-21.67). Carcinomas and epitheliomas differed from adenomas, with a Ki-67 index of 1.67% (0.00-5.00) and from the normal perianal glands, with an index of 0.00% (0.00-0.33) (P < 0.0001). Figure 4 shows the paired Ki-67 indexes for each tumor sample, obtained from both computer-assisted and manual counting, where it is possible to observe that, in spite of different numerical values, the methods gave more consistent results in adenomas than in carcinomas. In addition, the manual counting performed by two observers strongly agreed with each other but despite rather similar results, the data resulting from the computer-assisted image analysis were more homogeneous and obtained in a reliable and less time-consuming manner, compared to the data resulting from the manual counting.

**Figure 5:**
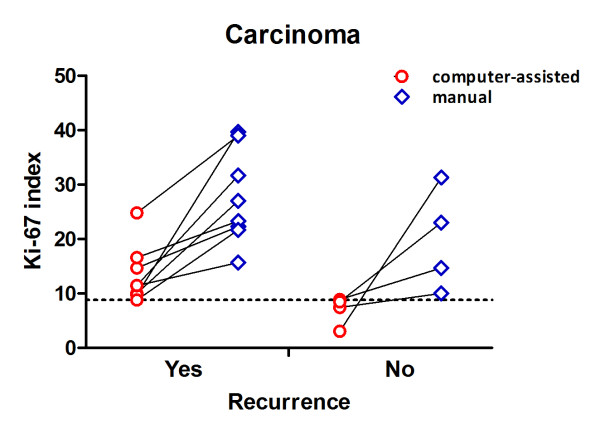
**Comparisons between the computer-assisted counting and the manual counting to establish the Ki-67 index in perianal gland carcinomas, taking into account the recurrence of the neoplasm.** The lines connect the values of each method in the same dog. The cut-off value (9.305) was obtained from a ROC-curve based on the data from the computer-assisted counting (area under the curve = 0.969; P = 0.0108).

Further, eight out of 12 dogs with carcinoma presented a historical report of recurrence after the surgical therapy. Using computer-assisted image analysis, a significantly (P = 0.0081) larger number of Ki-67 positive nuclei were detected, when comparing with those without recurrence (Figure 5). Despite using a small population, by means of a ROC curve, it was possible to establish the cut-off value of 9.305 (area under the curve = 0.969; P = 0.0108) to separate the perianal gland carcinomas that recurred from those without report of recurrence. Also, three out of 15 post-surgical recurrences were referenced for cases diagnosed as epitheliomas and three out of 15 for adenomas (Additional file 1: Table S1). In those cases, neither computer-assisted nor manual counting was effective to distinguish the dogs with or without evidence of recurrence.

## Discussion

Proliferation is a key feature of tumor progression and Ki-67 staining is a convenient method for assessing cell proliferation, applicable in most laboratories for humans [15,17] and animals [12-14]. The results presented here show that Ki-67 detection reflects the proliferation index of each type of perianal gland tumor. The Ki-67 index decreased from malignant towards benign lesions, with carcinomas presenting the highest Ki-67 index, which was useful to help the classification of the neoplasms, at least in less-differentiated samples. These findings are in agreement with previous studies using AgNOR (argyrophilic proteins related to nucleolar organizer regions) [18]. Similar to Ki-67, AgNOR is related to the proliferative activity of neoplasms and it may be associated with aggressive biological behavior – however, although the number of AgNORs increases in malignancy, the ability of this marker to define malignancy is still controversial [19].

In the routine diagnosis of perianal gland tumors, the use of HE staining generally allows an accurate diagnosis and the correct classification of adenomas, epitheliomas and carcinomas. However, in epitheliomas, areas with elevated anaplasia were frequently observed, suggesting transition to high-grade carcinomas. The use of proliferation markers, such as Ki-67, may help to confirm these differences and refine diagnostic criteria, particularly for well-differentiated perianal gland carcinomas [1].

Data relative to gender, age range, evolution time, and recurrence are in conformity with the available literature about perianal glands neoplasms in dogs [1]. Recurrence was related in eight out of 12 dogs with perianal gland carcinomas, in three dogs with epitheliomas, and three with adenomas. This disagreement among diagnosis and tumor behavior was previously described by Berrocal et al. [20], who observed recurrence in six out of 23 cases designated as “moderately or poorly differentiated” perianal gland adenomas, which included epitheliomas as well as low-grade carcinomas. Moreover, the recurrence in adenomas and epitheliomas may also have occurred due to neoplastic tissue remaining even after the surgical excision of the tumor. Regarding carcinomas, the samples with the highest Ki-67 indexes were taken from dogs that presented tumor recurrence, allowing us to suggest that the Ki-67 index, assessed by computer-assisted image analysis, may be used as a predictor of risk of recurrence, as also observed in other neoplasms [21-23].

Currently, there is a widely accepted use of Ki-67 to evaluate cell proliferation in cancer, but there is no standard protocol. One important factor that could bias the determination of the Ki-67 index is the subjective determination of the Ki-67 immunostained area. In the recent recommendations from the International Ki-67 in Breast Cancer Working Group [24], the authors stated that the Ki-67 evaluation method can be based on three patterns of Ki-67 immunostaining: homogeneous, hot spots, and a gradient of increasing staining toward the tumor edge; however, the most commonly used method to calculate the Ki-67 index is based on the hot spots, which also includes studies of prognostic value [12-14,22]. The recommendations from the same group [24] classified as ‘unknown’ which Ki-67 scoring method, manual (visual) or computer-assisted (automated), is superior. Despite being different types of neoplasms, based on the results presented herein, we provide evidence that the computer-assisted method is superior, at least in canine perianal gland tumors.

## Conclusions

Finally, this study established that the immunohistochemical detection of Ki-67 may help the morphological classification of more complex canine perianal gland neoplasms and it is helpful to better predict the risk of recurrence. The computer-assisted image analysis was demonstrated to be more accurate and a less time-consuming technique to evaluate the Ki-67 index, especially by facilitating the analysis where there is cell overlap or accumulation, when compared to manual counting. These results strongly suggest that a higher proliferation index is related to the aggressiveness of neoplastic cells and these differences must be taken into account to achieve a diagnosis and to establish the best therapeutic protocol.

## Competing interests

None of the authors has any financial or personal relationships that could inappropriately influence or bias the content of the paper.

## Authors’ contributions

RSP participated in the design of the study, carried out the immunohistochemical reactions, and helped to draft the manuscript. AS carried out the immunohistochemical reactions and participated in the computer-assisted image analysis. GDM participated in the computer-assisted image analysis, performed the statistical analysis, and helped to draft the manuscript. FVF carried out the immunohistochemical reactions. FARS collected the samples and the patients’ information. GFM conceived of the study, participated in its design and coordination, and helped to draft the manuscript. All authors read and approved the final manuscript.

## Supplementary Material

Additional file 1: Table S1 Individual data from each dog included in the experimental groups. **Figure S1.** Scatter plot showing the Ki-67 index assessed by manual counting, in different types of canine perianal gland neoplasms: adenoma (n = 15), epithelioma (n = 15), and carcinoma (n = 12), and in control dogs (n = 13). Horizontal lines indicate the median and the interquartile range. ^abc^ Groups with no common superscript letter differ significantly by Dunn’s multiple comparison test (P < 0.05). Click here for file

## References

[B1] Gross TL Ihrke P Walder EJ Affolter VK Blackwell O Gross TL, Ihrke P, Walder EJ, Affolter VK, Blackwell O Sebaceous tumors Skin diseases of the Dog and Cat. Clinical and Histopathologic Diagnosis 2005 2 Oxford: Blackwell Science 625 648

[B2] Withrow SJ Withrow SJ, Macewen EG Perianal tumors Small animal clinical oncology 2001 3 Philadelphia: Saunders 346 353

[B3] Yager JA Scott DW Jubb KVF, Kennedy PC, Palmer N The skin and appendages Pathology of domestic animals 1993 4 San Diego: Academic 531 738

[B4] Goldschmidt MH Hendrick MJ Meuten DJ Tumors of the skin and soft tissues Tumors in domestic animals 2002 4 Iowa: Ames 44 117

[B5] Ganguly A Wolfe LG Canine perineal gland carcinoma-associated antigen defined by monoclonal antibodies Hybridoma 2006 25 10 14 10.1089/hyb.2006.25.10 16475876

[B6] Pulley LT Stannard AA Moulton JE Tumors of the skin and soft tissues Tumors in domestic animals 1990 Berkeley: University of California 23 87

[B7] Goldschmidt MH Dunstan RW AAS Von Tscharner C Walder EJ Yager JA Histological Classification of Epithelial and Melanocytic Tumors of the Skin of Domestic Animals 1998 2 Washington: Armed Forces Institute of Pathology

[B8] Walder EJ Gross TL Gross TL, Ihrke PJ, Walder EJ Epithelial tumors Veterinary Dermatopathology: A macroscopic and microscopic evaluation of canine and feline skin disease 1992 Boston: Mosby 329 520

[B9] Gerdes J Schlueter CLL Duchrow M Wohlenberg C Gerlach C Stahmer I Kloth S Brandt E Flad H Immunobiochemical and molecular biologic characterization of the cell proliferation associated nuclear antigen that is defined by monoclonal antibody Ki-67 Am J Pathol 1991 138 867 873 2012175 PMC1886092

[B10] Taneja P Maglic D Kai F Zhu S Kending RD Fry EA Inoue K Classical and novel prognostic markers for breast cancer and theis clinical significance Clin Med Insights Oncol 2010 4 15 34 20567632 10.4137/cmo.s4773 PMC2883240

[B11] Brown D Gatter K Ki67 protein: the immaculate deception? Histopathology 2002 40 2 11 10.1046/j.1365-2559.2002.01343.x 11903593

[B12] Bergin IL Smedley RC Esplin DG Spangler WL Kiupel M Prognostic Evaluation of Ki67 Threshold Value in Canine Oral Melanoma Vet Pathol 2011 48 1 41 53 10.1177/0300985810388947 21123859

[B13] Peña LL Nieto AI Pérez-Alenza D Cuesta P Castaño M Immunohistochemical Detection of Ki-67 and PCNA in Canine Mammary Tumors: Relationship to Clinical and Pathologic Variables J Vet Diagn Invest 1998 10 3 237 246 10.1177/104063879801000303 9683072

[B14] Webster JD Yuzbasiyan-Gurkan V Miller RA Kaneene JB Kiupel M Cellular Proliferation in Canine Cutaneous Mast Cell Tumors: Associations with c-KIT and Its Role in Prognostication Vet Pathol 2007 44 3 298 308 10.1354/vp.44-3-298 17491070

[B15] Oliveira AM Sebo TJ McGrory JE Gaffey TA Rock MG Nascimento AG Extraskeletal myxoid chondrosarcoma: a clinicopathologic, immunohistochemical, and ploidy analysis of 23 cases Mod Pathol 2000 13 8 900 908 10.1038/modpathol.3880161 10955458

[B16] Melo GD Machado GF Glial reactivity in dogs with visceral leishmaniasis: correlation with T lymphocyte infiltration and with cerebrospinal fluid anti-*Leishmania* antibody titres Cell Tissue Res 2011 346 3 293 304 10.1007/s00441-011-1290-7 22160561

[B17] Urruticoechea A Smith IE Dowsett M Proliferation marker Ki-67 in early breast cancer J Clin Oncol 2005 23 7212 7220 10.1200/JCO.2005.07.501 16192605

[B18] Preziosi R Della Salda L, Ricci P, Sikoni P, Marcato PS: Quantification of nuclear organiser regions in canine perianal gland tumours Res Vet Sci 1995 58 277 281 10.1016/0034-5288(95)90117-5 7659856

[B19] Sivrides E Anastasiadis P Von Ludinghausen M Argyrophilic staining for nucleolar organizer region (AgNOR). A suitable methodology for differential diagnosis of breast lesions? Zentralbl Pathol 1992 138 2 103 107 1610761

[B20] Berrocal A Vos JH Van Den Ingh TS Molenbeek RF Van Sluljs FJ Canine perineal tumours Zentralbl Veterinarmed 1989 36 739 749 10.1111/j.1439-0442.1989.tb00787.x 2515683

[B21] Abry E Thomassen IØ Salvesen ØO Torp SH The significance of Ki-67/MIB-1 labeling index in human meningiomas: A literature study Pathol Res Pract 2010 206 12 810 815 10.1016/j.prp.2010.09.002 20951502

[B22] Bettencourt M-C Bauer JJ Sesterhenn IA Mostofi FK McLeod DG Moul JW Ki-67 Expression is a Prognostic Marker of Prostate Cancer Recurrence after Radical Prostatectomy J Urol 1996 156 3 1064 1068 10.1016/S0022-5347(01)65703-3 8709308

[B23] Valente G Orecchia R Gandolfo S Araudo M Ragona R Kerin S Palestro G Can Ki-67 immunostaining predict response to radiotherapy in oral squamous cell carcinoma? J Clin Pathol 1994 47 109 112 10.1136/jcp.47.2.109 8132823 PMC501821

[B24] Dowsett M Nielsen TO A’Hern R Bartlett J Coombes RC Cuzick J Ellis M Henry NL Hugh JC Lively T Assessment of Ki67 in Breast Cancer: Recommendations from the International Ki67 in Breast Cancer Working Group J Natl Cancer Inst 2011 103 22 1656 1664 10.1093/jnci/djr393 21960707 PMC3216967

