# Supplementary material for: Ki-67 labeling in canine perianal glands neoplasms: a novel approach for immunohistological diagnostic and prognostic
Source: BMC Vet Res. 2013 Apr 20;9:83. doi: 10.1186/1746-6148-9-83 (PMC3639849; doi:10.1186/1746-6148-9-83)
Supplement: Additional file 1: Table S1 — Individual data from each dog included in the experimental groups. Figure S1. Scatter plot showing the Ki-67 index assessed by manual counting, in different types of canine perianal gland neoplasms: adenoma (n = 15), epithelioma (n = 15), and carcinoma (n = 12), and in control dogs (n = 13). Horizontal lines indicate the median and the interquartile range. abc Groups with no common superscript letter differ significantly by Dunn’s multiple comparison test (P < 0.05). [file 1746-6148-9-83-S1.docx]

**Additional file 1.**

**Table S1.** Individual data from each dog included in the experimental groups.

| **ID #** | **Group** | **Sex** | **Age (years)** | **Breed** | **Spaying** | **Follow-up (months)¹** | **Recurrence** | **Ki-67 index²** | |
| --- | --- | --- | --- | --- | --- | --- | --- | --- | --- |
|  |  |  |  |  |  |  |  | **Computer-assisted** | **Manual counting** |
| 001/08 | Adenoma | F | 7 | Shitzu | Yes | 12 | Yes | 0.24 | 1.33 |
| 075/08 | Adenoma | M | 9 | Mongrel | No | 24 | No | 0.22 | 1.00 |
| 1101/07 | Adenoma | M | 9 | Siberian husky | No | 9 | Yes | 1.43 | 4.33 |
| 1188/06 | Adenoma | M | 9 | Poodle | No | 12 | No | 1.08 | 5.00 |
| 1253/07 | Adenoma | F | 5 | Maltese | Yes | 24 | Yes | 0.00 | 0.00 |
| 1392/07 | Adenoma | M | 8 | Poodle | No | 18 | No | 0.56 | 1.67 |
| 1708/07 | Adenoma | M | 11 | Poodle | No | 12 | No | 0.15 | 0.67 |
| 1772/05 | Adenoma | M | 8 | Siberian husky | No | 12 | No | 0.00 | 0.00 |
| 260/08 | Adenoma | M | 13 | Poodle | No | 12 | No | 0.78 | 2.00 |
| 632/08 | Adenoma | F | 8 | Cocker spaniel | Yes | 8 | No | 0.00 | 0.00 |
| 634/07 | Adenoma | M | 7 | Akita | No | 24 | No | 0.27 | 1.00 |
| 676/08 | Adenoma | F | 12 | Poodle | No | 24 | No | 0.41 | 1.67 |
| 713/05 | Adenoma | M | 9 | English bulldog | No | 24 | No | 0.36 | 1.67 |
| 835/08 | Adenoma | M | 10 | Siberian husky | No | 10 | No | 0.60 | 2.67 |
| 980/08 | Adenoma | M | 11 | Mongrel | No | 24 | No | 0.51 | 2.33 |
| 1148/07 | Epithelioma | M | 15 | Beagle | No | 6 | No | 5.56 | 21.67 |
| 1181/07 | Epithelioma | M | 10 | Mongrel | Yes | 10 | No | 2.66 | 12.67 |
| 1222/06 | Epithelioma | M | 10 | Rottweiler | No | 24 | Yes | 1.17 | 3.67 |
| 1304/06 | Epithelioma | M | 13 | Dachshund | No | 12 | No | 2.42 | 8.67 |
| 1365/07 | Epithelioma | M | 10 | Lhasa Apso | No | 12 | No | 1.94 | 7.00 |
| 1447/07 | Epithelioma | M | 8 | Akita | No | 24 | Yes | 3.72 | 18.67 |
| 1679/07 | Epithelioma | M | 11 | Mongrel | No | 12 | No | 1.41 | 6.00 |
| 189/07 | Epithelioma | M | 10 | Mongrel | No | 12 | No | 7.10 | 14.33 |
| 236/07 | Epithelioma | M | 11 | Beagle | No | 12 | Yes | 4.02 | 11.33 |
| 534/06 | Epithelioma | M | 13 | Pointer | No | 24 | No | 0.54 | 3.00 |
| 657/07 | Epithelioma | M | 13 | Cocker spaniel | Yes | 24 | No | 2.70 | 8.33 |
| 668/06 | Epithelioma | M | 10 | Poodle | No | 36 | No | 2.31 | 6.67 |
| 704/08 | Epithelioma | F | 8 | Mongrel | No | 24 | No | 4.31 | 9.67 |
| 858/06 | Epithelioma | M | 11 | Poodle | No | 12 | No | 5.10 | 14.67 |
| 873/06 | Epithelioma | M | 10 | Mongrel | No | 8 | No | 2.06 | 15.00 |
| 138/06 | Carcinoma | M | 8 | Mongrel | No | 12 | No | 8.47 | 23.00 |
| 197/05 | Carcinoma | M | 10 | Mongrel | No | 12 | Yes | 8.89 | 21.67 |
| 216/08 | Carcinoma | F | 3 | Mongrel | Yes | 4 | Yes | 24.82 | 39.00 |
| 296/06 | Carcinoma | M | 5 | Poodle | No | 6 | Yes | 14.68 | 22.33 |
| 521/06 | Carcinoma | M | 12 | Dachshund | No | 24 | Yes | 11.43 | 15.67 |
| 554/06 | Carcinoma | M | 6 | Poodle | No | 9 | No | 8.90 | 14.67 |
| 566/07 | Carcinoma | M | 11 | Fox Terrier | No | 12 | No | 7.45 | 10.00 |
| 677/06 | Carcinoma | M | 11 | Mongrel | No | 5 | Yes | 16.59 | 23.33 |
| 678/06 | Carcinoma | M | 10 | Siberian husky | No | 12 | Yes | 11.48 | 31.67 |
| 688/06 | Carcinoma | F | 6 | Basset hound | No | 12 | No | 3.09 | 31.33 |
| 688/07 | Carcinoma | F | 8 | Cocker spaniel | No | 12 | Yes | 10.03 | 27.00 |
| 831/06 | Carcinoma | M | 10 | Akita | No | 7 | Yes | 9.71 | 39.67 |
| C1 | Control | F | 3 | Mongrel | No | - | - | 0.00 | 0.00 |
| C2 | Control | F | 10 | Mongrel | No | - | - | 0.00 | 0.00 |
| C3 | Control | F | 8 | Poodle | No | - | - | 0.00 | 0.00 |
| C4 | Control | M | 9 | Mongrel | No | - | - | 0.01 | 0.33 |
| C5 | Control | F | 10 | Mongrel | No | - | - | 0.00 | 0.00 |
| C6 | Control | M | 5 | Mongrel | No | - | - | 0.00 | 0.00 |
| C7 | Control | M | 9 | Mongrel | No | - | - | 0.00 | 0.00 |
| C8 | Control | M | 6 | Mongrel | No | - | - | 0.00 | 0.00 |
| C9 | Control | M | 8 | Akita | No | - | - | 0.00 | 0.00 |
| C10 | Control | F | 10 | Mongrel | No | - | - | 0.00 | 0.00 |
| C11 | Control | M | 3 | Mongrel | No | - | - | 0.00 | 0.00 |
| C12 | Control | F | 12 | Mongrel | No | - | - | 0.07 | 0.33 |
| C13 | Control | M | 9 | Mongrel | No | - | - | 0.02 | 0.33 |

¹ time elapsed from the detection of the perianal nodule by the owner to the surgery (nodulectomy)

² median values


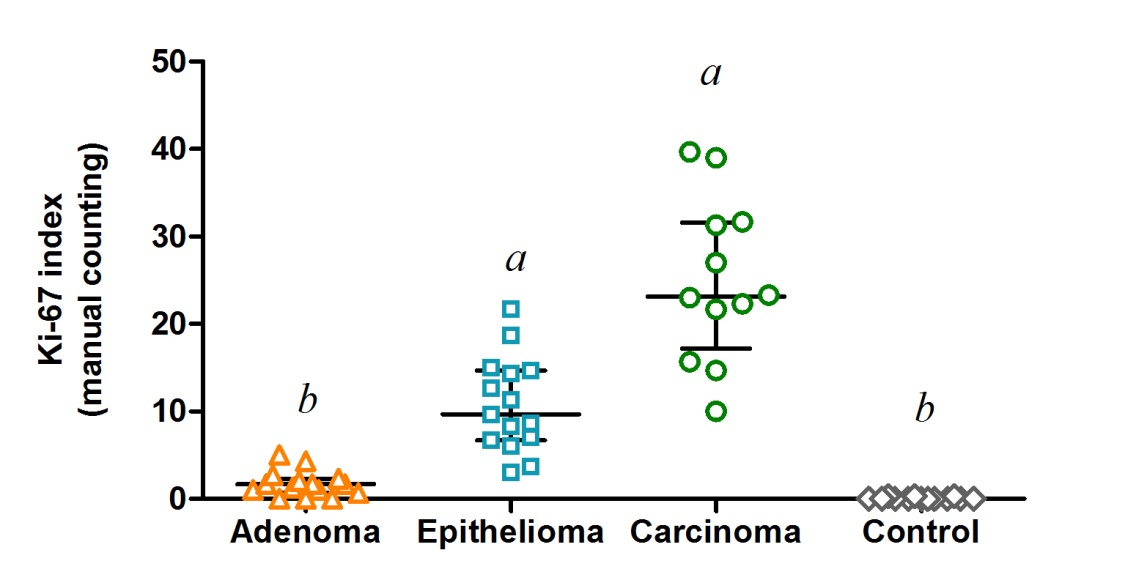


**Figure S1.** Scatter plot showing the Ki-67 index assessed by manual counting, in different types of canine perianal gland neoplasms: adenoma (n=15), epithelioma (n=15), and carcinoma (n=12), and in control dogs (n=13). Horizontal lines indicate the median and the interquartile range. ^abc^ Groups with no common superscript letter differ significantly by Dunn’s multiple comparison test (P<0.05).
